# The Biofilm Lifestyle Shapes the Evolution of β-Lactamases

**DOI:** 10.1093/gbe/evae030

**Published:** 2024-02-15

**Authors:** Øyvind M Lorentzen, Anne Sofie B Haukefer, Pål J Johnsen, Christopher Frøhlich

**Affiliations:** Department of Pharmacy, UiT The Arctic University of Norway, Tromsø, Norway; Department of Pharmacy, UiT The Arctic University of Norway, Tromsø, Norway; Department of Pharmacy, UiT The Arctic University of Norway, Tromsø, Norway; Department of Pharmacy, UiT The Arctic University of Norway, Tromsø, Norway

**Keywords:** *Vibrio cholerae*, β-lactamases, evolution, AMR, biofilm

## Abstract

The evolutionary relationship between the biofilm lifestyle and antibiotic resistance enzymes remains a subject of limited understanding. Here, we investigate how β-lactamases affect biofilm formation in *Vibrio cholerae* and how selection for a biofilm lifestyle impacts the evolution of these enzymes. Genetically diverse β-lactamases expressed in *V. cholerae* displayed a strong inhibitory effect on biofilm production. To understand how natural evolution affects this antagonistic pleiotropy, we randomly mutagenized a β-lactamase and selected for elevated biofilm formation. Our results revealed that biofilm evolution selects for β-lactamase variants able to hydrolyze β-lactams without inhibiting biofilms. Mutational analysis of evolved variants demonstrated that restoration of biofilm development was achieved either independently of enzymatic function or by actively leveraging enzymatic activity. Taken together, the biofilm lifestyle can impose a profound selective pressure on antimicrobial resistance enzymes. Shedding light on such evolutionary interplays is of importance to understand the factors driving antimicrobial resistance.

## Graphical Abstract

**Graphical abstract:**
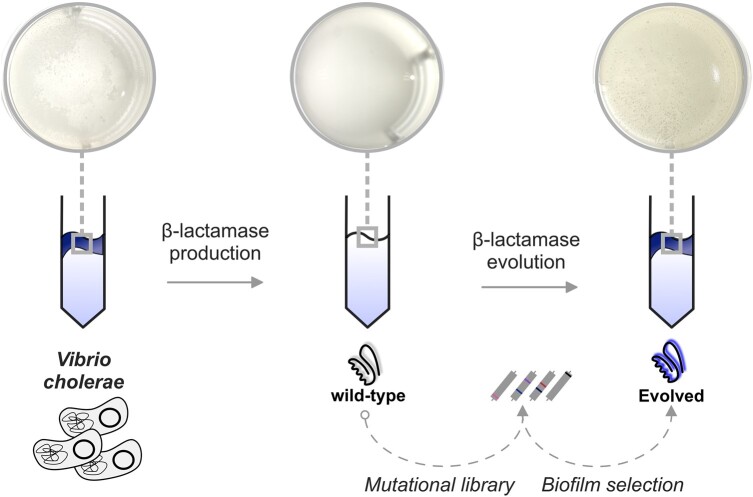

**Significance**

β-Lactamases inhibit biofilm formation, and the selection for increased biofilm production can mitigate this antagonistic pleiotropic effect. The emergence of β-lactamase variants avoiding biofilm inhibition suggests that the biofilm lifestyle affects the evolutionary fate of these enzymes.

## Introduction

Biofilms, which are structured bacterial communities covered in a protective extracellular matrix, represent one of the most prevalent bacterial lifestyles (Flemming et al. 2016; Ciofu et al. 2022). Biofilm-embedded bacteria demonstrate a remarkable ability to endure harsh conditions and exhibit increased tolerance toward external stressors, including antimicrobials (Flemming et al. 2016; Ciofu et al. 2022). These structured communities further serve as hotspots that facilitate the dissemination of mobile genetic elements harboring antimicrobial resistance genes (Madsen et al. 2012; Abe et al. 2020; Castañeda-Barba et al. 2023). It has been shown that a biofilm lifestyle can select for distinct evolutionary trajectories and profoundly influences the evolution of both bacteria and mobile genetic elements, when compared to bacteria evolving in unstructured environments (Steenackers et al. 2016; Kovács and Dragoš 2019; Coenye et al. 2022; Castañeda-Barba et al. 2023). However, our current understanding of how biofilms influence the evolution of antimicrobial resistance enzymes is limited.

Upon acquisition, plasmid-harbored genes can induce pleiotropy, resulting in unpredictable effects on multiple cellular traits such as reduced basal bacterial growth or collateral responses to antimicrobials (Baltrus et al. 2021; Billane et al. 2021; Roemhild et al. 2022). Consequently, pleiotropy plays a pivotal role in shaping natural selection in a given environment, potentially requiring compensatory mutations to counteract these adverse effects.

Among Gram-negative pathogens, the most prominent cause of β-lactam resistance is the production of β-lactamases (Cassini et al. 2019). These enzymes display significant sequence- and functional-variability and are often encoded on mobile genetic elements, which facilitates horizontal transmission to closely and more distantly related bacteria (Castañeda-Barba et al. 2023). They can be classified into Ambler classes A to D based on sequence diversity or grouped into 2 major functional categories: serine-type (classes A, C, and D) and metallo-β-lactamases (class B) (Bush 2018). Enzymes grouped into classes A and D have been shown to antagonize biofilm formation in *Escherichia coli* and *Pseudomonas aeruginosa* (Gallant et al. 2005; Fernández et al. 2012). We hypothesize that the occurrence of such pleiotropic effects can significantly alter the evolutionary trajectory of the pleiotropy-inducing resistance enzymes.

**Fig. 1.**
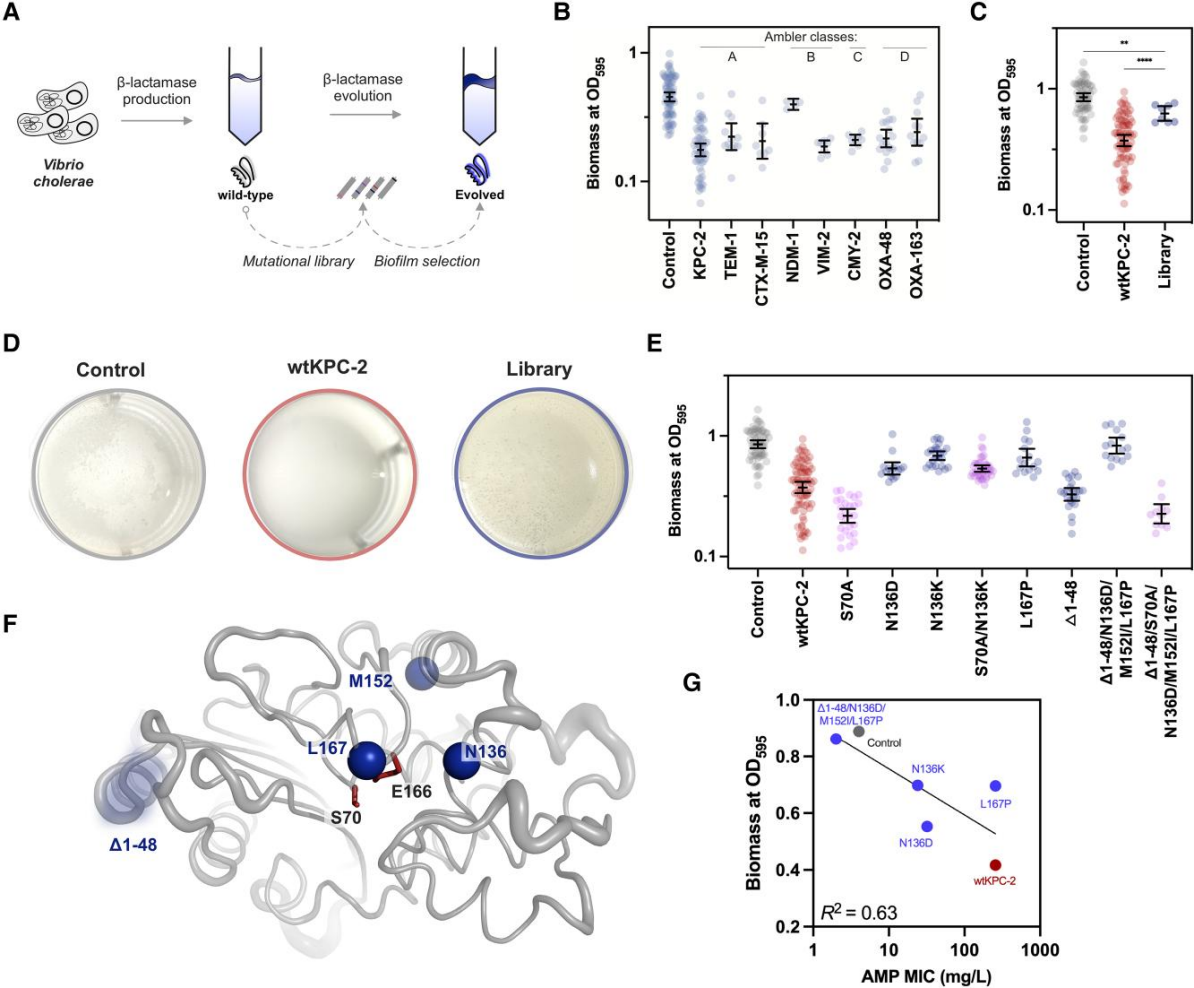
Biofilm lifestyle shapes the evolution of β-lactamases in *V. cholerae*. a. We first explored the influence of β-lactamase gene expression on the *V. cholerae* biofilm phenotype (left). Second, by subjecting a mutant library of KPC-2 to experimental evolution, we revealed how the biofilm lifestyle affects β-lactamase evolution (right). b. The expression of β-lactamase genes from Ambler classes A to D (top) significantly hindered biofilm formation in *V. cholerae* compared to the control vector. c. Our mutational library of KPC-2 (>5,000 mutants) exhibited significantly enhanced biofilm formation compared to wild-type KPC-2 (wtKPC-2) (****; one-way ANOVA, *P* < 0.0001), although it remained less than the vector control (**; one-way ANOVA, *P* = 0.001). d. Differences in biomass production related to *V. cholerae*'s ability to form pellicles. While our control displayed signs of pellicle formation after 24 h incubation, the presence of KPC-2 completely suppressed biofilm pellicle development. In contrast, the presence of our KPC-2 mutational library resulted in a well-structured biofilm pellicle. e. While wtKPC-2 led to reduced biofilm capacity, N136K and Δ1-48/N136D/M152I/L167P, which were selected from and were enriched in the pellicle, demonstrated significant improvement in biofilm formation (*P* values reported in Table 2). β-Lactam binding-deficient (serine-to-alanine at position 70) variants of wtKPC-2 and Δ1-48/N136D/M152I/L167P strongly reduced the biofilm phenotype. On the contrary, S70A/N136K maintained high levels of biofilm formation compared to the evolved variant N136K. Deconvolution of mutations within Δ1-48/N136D/M152I/L167P displayed that, in contrast to N136D and L167P, the deletion Δ1-48 did not significantly increase biofilm formation compared to wtKPC-2 (*P* values reported in Table 2). f. Location of mutational sites compared to the key active site residues S70 and E166. g. Pearson correlation (*R*² = 0.63, *P* = 0.059) between ampicillin resistance and biofilm formation for wtKPC-2 (black), control (gray), and evolved mutants Δ1-48/N136D/M152I/L167P, N136K, N136D, and L167P (blue). Each datapoint in b, c, and e represents a biological replicate, and error bars display 95% confidence intervals.

In this study, we utilize *Vibrio cholerae* as a model organism, due to the importance of biofilm in its life cycle, to study the impact of β-lactamases on biofilm formation (Teschler et al. 2015; Conner et al. 2016; Silva and Benitez 2016; Teschler et al. 2022). We employed a combination of directed and experimental evolution techniques (Fig. 1a) to evaluate how selection for pellicle production, a specific type of biofilm formed at the air-liquid interface, influences the evolutionary trajectories of β-lactamases (Kovács and Dragoš 2019; Qin et al. 2021; Qin and Bassler 2022). Gaining insights into these intricate evolutionary relationships is essential for comprehending the dissemination and evolution of antimicrobial resistance enzymes.

## Results and Discussion

### β-Lactamases From All Ambler Classes Inhibit Biofilm Formation

To determine the inhibitory effect of β-lactamases on biofilm formation, we quantified biomass produced by *V. cholerae* strains harboring a medium copy number vector with or without β-lactamase genes (Fig. 1a and b). Crystal violet staining of adherent biomass after 24 h of static growth was used as a proxy for biofilm development. Compared to the control vector, 7 out of 8 tested β-lactamase-producing strains exhibited a significant reduction in biomass ranging from 43% to 61% (Table 1 and Fig. 1b, one-way analysis of variance [ANOVA], *P* < 0.0001). Notably, NDM-1 was the only exception, showing a statistically nonsignificant reduction of 17% (Table 1 and Fig. 1b, one-way ANOVA, *P* > 0.05). While it has been previously suggested that biofilm inhibition is mainly attributed to class A and D β-lactamases, due to their evolutionary relationship to low-molecular-weight penicillin-binding proteins (Gallant et al. 2005; Fernández et al. 2012), our data demonstrate that this antagonistic pleiotropy can be more general across the different classes of β-lactamases.

**Table 1:** Biofilm inhibitory effect of β-lactamases from all Ambler classes

Strain no.	β-Lactamases	Ambler class	Biomass at OD_595_^a^	*P* values^b^	*N* ^c^	Relative fitness^d^	Ampicillin MICs (mg/L)
30-73	wtVC^e^	—	0.800 ± 0.021	<0.0001	10	1.48 ± 0.01	4
30-71	Control^f^	—	0.483 ± 0.019	—	77	1	4
30-70	KPC-2	A	0.191 ± 0.011	<0.0001	56	1.34 ± 0.01	>256
32-56	TEM-1	A	0.238 ± 0.028	0.0002	12	0.80 ± 0.01	24
32-57	CTX-M-15	A	0.220 ± 0.034	0.0036	8	1.22 ± 0.01	>256
32-58	NDM-1	B	0.399 ± 0.012	0.1397	4	1.07 ± 0.1	>256
32-59	VIM-2	B	0.189 ± 0.008	<0.0001	8	1.26 ± 0.01	>256
32-53	CMY-2	C	0.211 ± 0.008	<0.0001	8	0.94 ± 0.03	32
32-54	OXA-48	D	0.225 ± 0.016	<0.0001	16	1.32 ± 0.01	>256
32-55	OXA-163	D	0.259 ± 0.030	0.0008	12	0.97 ± 0.03	>256

Errors are reported as the standard error of the mean.

MIC, minimal inhibitory concentration; OD, opitcal density.

^a^OD measurements were performed in a 96-well plate.

^b^Brown–Forsythe one-way ANOVA to test for differences in biomass formation compared to the vector control (*α* = 0.05) and followed by Dunnett test to correct for multiple testing; F (DFn, DFd) = 48.50 (8, 77); overall *P* < 0.0001.

^c^Sample size (biological replicates) tested to determine the biomass at OD_595_.

^d^Determined as area under the growth curve compared to control (see supplementary fig. S1, Supplementary Material online for bacterial growth curves and supplementary table S1, Supplementary Material online for areas under curve).

^e^Wild-type *V. cholerae* C6706. Parental strain for all downstream strain constructs.

^f^ *V. cholerae* harboring the pA15 vector without β-lactamase gene.

To ensure that the expressed β-lactamases were functional, we determined the ampicillin resistance and bacterial fitness of the β-lactamase-producing strains. Our data show that the β-lactamases conferred a 6- to 64-fold decrease in ampicillin susceptibility compared to our vector control, confirming the functional activity of the enzymes (Table 1). Evaluation of the area under the growth curve, used as a proxy for bacterial fitness, uncovered that 7 out of 8 β-lactamase-producing strains did not suffer a detrimental effect on fitness (Table 1, supplementary fig. S1, Supplementary Material online, and supplementary table S1, Supplementary Material online). Unexpectedly, the TEM-1-producing strain demonstrated reduced fitness (20%) compared to the empty vector control strain. The same strain conferred only a 6-fold increase in ampicillin minimal inhibitory concentration (MIC), despite the overall high catalytic activity of TEM-1 (*k*_cat_/*K*_M_ ∼10^7^ M^−1^ s^−1^) (Brown et al. 2009). Therefore, in the case of TEM-1, it cannot be excluded that the observed mediated biofilm inhibition was unrelated to the intracellular enzyme production. However, correlation analysis of the relationship bacterial fitness of all β-lactamase-producing strains and their ability to produce biofilms did not reveal any significant correlation (Pearson correlation, *R*² = 0.15, *P* = 0.31; supplementary fig. S1, Supplementary Material online). Thus, the overall antagonization of biofilms mediated by β-lactamases was likely not attributable to nonfunctional proteins or detrimental effects on bacterial fitness but appeared to be connected to their enzymatic activity.

### The Biofilm Lifestyle Shapes the Evolution of β-Lactamases

To understand whether genetic changes within β-lactamases could modulate biofilm formation, we focused on the contemporary β-lactamase KPC-2 (wtKPC-2), as it conferred a strong biofilm inhibitory effect, and employed random mutagenesis as a mean to generate genetic diversity (Fig. 1a). Expression of the gene library in *V. cholerae* significantly improved biofilm formation, resulting in higher biomass (OD_595_) compared to the wtKPC-2 (Fig. 1c, one-way ANOVA, *P* < 0.0001). Improvements were also evident in the strains’ ability to form biofilm pellicles (Fig. 1d). The presence of wtKPC-2 completely suppressed pellicle formation, while both the control and mutant library formed visible biofilm pellicles at the air-liquid interface. Thus, our results indicate that the library harbors *bla*_KPC-2_ mutants able to compensate for the initial biofilm antagonization.

To identify potential variants displaying compensatory behavior, we harvested the biofilm pellicles formed by the library population-mix (*n* = 2) and isolated 12 random clones. Sanger sequencing revealed that 33% of the selected clones contained mutations within KPC-2 at position 136 (N136K and N136D), indicating strong selection for pellicle production and parallel evolution. The clone displaying N136D also exhibited additional amino acid substitutions (M152I/L167P) and a deletion which led to a frameshift mutation (supplementary fig. S2, Supplementary Material online). This frameshift resulted in the loss of the first 48 amino acids, including the signal peptide, and the recruitment of an alternative methionine start codon at position 49 (Δ1-48/N136D/M152I/L167P) (supplementary fig. S2, Supplementary Material online).

To remove the effect of potential confounding mutations on the vector backbone and chromosome, we subcloned N136K and Δ1-48/N136D/M152I/L167P into an isogenic vector backbone and assayed biofilm formation. N136K and Δ1-48/N136D/M152I/L167P displayed 68% and 107% higher biomass relative to wtKPC-2, respectively (one-way ANOVA, *P* < 0.0001 and *P* = 0.0008; Table 2 and Fig. 1e). To deconvolute the contributions from the different mutations in the Δ1-48/N136D/M152I/L167P variant, we selected and constructed variants displaying either loss of the signal peptide (Δ1-48) or mutations around the active site (N136D and L167P; Table 2 and Fig. 1e). While Δ1-48 alone did not significantly (one-way ANOVA, *P* = 0.79) improve biofilm formation relative to wtKPC-2, N136D and L167P increased biofilm formation by 33% (one-way ANOVA, *P* = 0.0003) and 67% (one-way ANOVA, *P* < 0.0001), respectively (Table 2 and Fig. 1e). Translocation of β-lactamases into the periplasmic space depends on the presence of a signal peptide, and the loss of a signal peptide (e.g. Δ1-48/N136D/M152I/L167P) prevents this. Thus, KPC-2 variants without this signal peptide are likely retained in the cytoplasm. Our data demonstrate that these mutants still exert a detrimental effect on biofilm formation. In addition, β-lactamases bearing a signal peptide (e.g. N136D/K and L167P) can be enzymatically active within the cytosol before the translocation (Paunola et al. 1998). This observation suggests that the mechanistic interaction by which they interfere with biofilm formation likely occurs in the cytoplasm. Taken together, our result shows that a biofilm lifestyle can select for mutations in β-lactamases that reverse their initial antagonistic pleiotropic effect on biofilm formation. Thus, evolution in biofilms shapes the evolution of antimicrobial resistance enzymes.

**Table 2:** KPC-2 variants resulting in improved biofilm development

Strain no.	Inserts	Biomass at OD_595_^a^	*P* values^b^	*N* ^c^	Relative fitness^d^	Ampicillin MICs (mg/L)
30-70	wtKPC-2	0.417 ± 0.020	—	81	1	>256
30-71	Control^e^	0.889 ± 0.032	<0.0001	61	0.75 ± 0.01	4
30-65	S70A	0.229 ± 0.014	<0.0001	27	ND	4
30-77	Δ1-48/N136D/M152I/L167P^e^	0.862 ± 0.063	<0.0001	15	0.78 ± 0.04	2
30-67	Δ1-/S70A/N136D/M152I/L167P**^e^**	0.235 ± 0.021	0.0008	11	ND	4
32-13	Δ1-48	0.341 ± 0.019	0.7914	24	0.79 ± 0.03	2
32-07	N136D	0.553 ± 0.037	0.0003	16	0.97 ± 0.02	32
30-75	N136K	0.698 ± 0.028	<0.0001	24	1.00 ± 0.01	24
30-69	S70A/N136K	0.547 ± 0.019	<0.0001	39	ND	2
32-06	L167P	0.696 ± 0.061	<0.0001	16	0.99 ± 0.01	>256

MIC, minimal inhibitory concentration; ND, not determined; OD, opitcal density.

^a^OD measurement was performed in 24-well plates.

^b^Brown–Forsythe one-way ANOVA to test for differences in biomass formation compared to wtKPC-2 (*α* = 0.05) and followed by Dunnett test to correct for multiple testing; F (DFn, DFd) = 62 (15, 293); overall *P* value < 0.0001.

^c^Sample size (biological replicates) tested to determine the biomass at OD_595_.

^d^Determined as area under the growth curve compared to wtKPC-2 (see supplementary fig. S3, Supplementary Material online for bacterial growth curves and supplementary table S1, Supplementary Material online for area under curves).

^e^ *V. cholerae* harboring the pA15 vector without β-lactamase gene.

Errors are reported as the standard error of the mean.

### Functional Mutations in KPC-2 Reverse Biofilm Inhibition

Next, we investigated the functional and structural role of mutations acquired during evolution. To study the functionality of selected and constructed mutants, we investigated their ability to confer ampicillin resistance (Table 2). As expected, all mutants lacking the signal peptide exhibited ampicillin susceptibility similar to the vector control (Table 2). Other mutations, such as N136D/K and L167P, clustered around the active site of KPC-2 (Fig. 1f) maintained ampicillin MICs 6- to >64-fold higher than the control strain. Generally, increased biofilm formation coincided with lower ampicillin resistance (Pearson correlation, *R*² = 0.63, *P* = 0.059). However, our L167P mutant maintained ampicillin susceptibility similar to KPC-2 while compensating for biofilm formation. L167P exemplifies that biofilm compensation can occur without compromising the enzyme's ability to confer ampicillin resistance (Fig. 1g). Furthermore, the fitness effect of these mutants did not significantly correlate with biofilm formation (Pearson correlation, *R*^2^ = 0.004 and *P* = 0.89, supplementary fig. S3, Supplementary Material online). Altogether, our combined findings on bacterial growth and biofilm formation indicate that the reversal of biofilm inhibition was not related to changes in bacterial fitness.

Our mutant analysis (Fig. 1e) suggests that the compensatory effects of the evolved mutants may be linked to cytosolic processes, where they could be either permanently active due to loss of the signal peptide (Δ1-48/N136D/M152I/L167P) or temporarily (N136D/K and L167P) prior to translocation (Paunola et al. 1998). To investigate whether the enzymatic activity of wtKPC-2 and evolved variants was linked to biofilm formation, we constructed serine-to-alanine mutants at position 70 which are unable to covalently bind and efficiently hydrolyze β-lactam substrates (Table 2 and Fig. 1e) (Stojanoski et al. 2016). As expected, introducing S70A in the wtKPC-2, N136K, and Δ1-48/N136D/M152I/L167P backgrounds resulted in MICs similar to the vector control (Table 2). Introducing S70A in wtKPC-2 led to a 45% reduction in biofilm formation compared to the wtKPC-2. Similarly, the introduction of S70A in the evolved Δ1-48/N136D/M152I/L167P variant caused a 73% decrease in biofilm formation compared to the evolved variant, resulting in biofilm levels similar to KPC-2:S70A (Fig. 1e). On the contrary, S70A/N136K displayed only a 21% reduction in biofilm formation compared to the evolved N136K variant and maintained a strong biofilm phenotype relative to the other serine-to-alanine mutants. Our findings stand in contrast to a previous study where the removal of the active site serine either rescued (TEM-1) or had no effect (OXA-3) on biofilm formation (Gallant et al. 2005). Therefore, how enzyme functionality affects biofilm inhibition seems to vary greatly between different β-lactamases. While the exact molecular mechanisms of biofilm antagonization and compensation remain elusive, informed by our data, we hypothesize that the evolved variants compensate for biofilm inhibition either independently of enzymatic activity (N136K) or by employing its enzymatic activity (Δ1-48/N136D/M152I/L167P). For Δ1-48/N136D/M152I/L167P, the reversal of biofilm inhibition seemingly relies on the indispensable active site serine (S70), since introducing S70A into Δ1-48/N136D/M152I/L167P nullifies the reversal. This indicates that Δ1-48/N136D/M152I/L167P relies on the enzymatic activity of KPC-2 (Fig. 1e) to increase biofilm formation. Alternatively, observed differences in biofilm formation between mutants may be related to differences in structural integrity and/or stability of the evolved variants. However, this seems unlikely since S70A usually does not compromise the stability of β-lactamases (Brown et al. 2009).

Taken together, our findings demonstrate that a broad range of highly diverse β-lactamases inhibits biofilm formation in *V. cholerae,* and that selection for a biofilm lifestyle significantly affects the evolution of these enzymes. Such pleiotropy, where genes can affect a multitude of bacterial phenotypes, has been observed in multiple model systems (Andersson and Hughes 2010; Noda-García et al. 2019; Burmeister et al. 2020). We argue that the selection pressure generated through pleiotropic effects represents a substantial selective force, which influences the genetic adaption and evolution of antimicrobial resistance enzymes.

## Methods and Material

### Growth Media and Chemicals

All strains and primers used and constructed within this study are shown in supplementary tables S2 and S3, Supplementary Material online. Strains were grown in Lysogeny-Broth (LB) media supplemented with chloramphenicol (5 or 25 mg/L for *V. cholerae* and *E. coli* strains, respectively). LB media and chloramphenicol were purchased from Sigma-Aldrich (USA). Restriction enzymes and T4 ligase were supplied by ThermoFisher (USA).

### Strain Construction

The gene sequences of *bla*_TEM-1_, *bla*_CMY-2_, *bla*_CTX-M-15_, and *bla*_NDM-1_ were previously synthesized by Genewiz (Germany) and subcloned in a medium copy number vector (p15A origin) according to the gene sequences NG_050145.1, NG_048935.1, NG_048814.1, and NG_049326.1, respectively (Fröhlich et al. 2022). In addition, *bla*_VIM-2_ (NG_050347.1), *bla*_OXA-48_ (CP033880), and *bla*_KPC-2_ (KU665642) were subcloned from *E. coli* 50579417 and *Klebsiella pneumoniae* K47-25, respectively, into the same vector backbone (Samuelsen et al. 2013; Di Luca et al. 2017; Taiaroa et al. 2018; Fröhlich et al. 2022). All β-lactamases carried an additional glycine after their start codon, allowing us to use a *Xho*I restriction site at the N-terminus. Amplification was performed with Phusion polymerase (NEB). PCR products were digested using *Dpn*I, *Xho*I, and *Nco*I and ligated with the backbone using T4 ligase. Ligated vectors were transformed into the *E. coli* E. cloni (MP21-5) and then subsequently transformed into *V. cholerae* C6706.

OXA-163 was constructed by site-directed mutagenesis and whole vector amplification using Phusion polymerase (NEB, USA), primers P54F/R, and *bla*_OXA-48_ (CP033880) as a template (Fröhlich et al. 2019). The PCR product was digested for 1 h at 37 °C using *Dpn*I and *Lgu*I. The digested product was ligated for 1 h at room temperature using T4 ligase and transformed into *E. coli* E. cloni (MP21-5). Cells were selected on chloramphenicol 25 mg/L, and mutations were confirmed with Sanger sequencing.

To subclone mutant *bla*_KPC-2_ alleles, the target genes and vector backbone were amplified using primers P7/P8 and P3/P4, respectively (supplementary table S3, Supplementary Material online), and Phusion polymerase (NEB). PCR products were digested using *Dpn*I, *Xho*I, and *Nco*I and ligated with the backbone using T4 ligase. Ligated vectors were transformed into the MP21-5 and then subsequently transformed into *V. cholerae* C6706.

Active site serine of KPC-2 was mutated to alanine (S70A) using whole vector site-directed mutagenesis with primers P108/P115 containing *Lgu*I cutting sites. The *bla*_KPC-2_ genes were amplified using primers P108/P115 and Phusion polymerase (NEB). The PCR products were digested with *Lgu*I and *Dpn*I for 1 h at 37 °C following self-ligation using T4 ligase. Ligated vectors were transformed into MP21-5 and then subsequently transformed into *V. cholerae* C6706.

### Bacterial Fitness Measurements

Single colonies were grown overnight at 37 °C with shaking at 700 rpm and subsequently diluted 1:100 into LB medium supplemented with 5 µg/L chloramphenicol to a final volume of 300 μL. Growth curve experiments were conducted in 100-well honeycomb plates in a Bioscreen C instrument (Oy Growth Curves Ab Ltd, Finland). Briefly, growth curves were recorded by measuring optical density at 600 nm (OD_600_) in 4 min intervals for 18 h at 37 °C with continuous shaking. The relative bacterial fitness was calculated as the area under the curve of the individual growth curves using the flux package in R (Jurasinski et al. 2014) and normalized to either *V. cholerae* harboring an empty control vector (Table 1) or wtKPC-2 (Table 2). Fitness was calculated based on a minimum of 3 biological replicates each based on 3 technical replicates per biological replicate.

### Biomass Determining Using Crystal Violet

Overnight cultures were grown in 2 mL LB medium supplemented with 5 µg/L chloramphenicol and incubated overnight at 37 °C with shaking (700 rpm). The following day, the cultures were diluted 1:100 in 2 mL LB medium in a 24-well plate (Corning, USA) and incubated statically at 37 °C for 24 h. Pellicle formation was imaged with a NexiusZoom stereo microscope (Euromex, Netherlands) at 6.7× magnification. Next, the bacterial cultures were removed from the 24-well plate, and the plate was gently washed in distilled water to remove non-adherent bacterial cells. Biofilms were fixed by incubation for 1 h at 55 °C. To quantify the attached biomass, cells were stained with 2 mL of 0.1% crystal violet (Sigma-Aldrich) for 10 min. The crystal violet solution was then removed, and the plates were washed in filtered water and dried at room temperature. Crystal violet-stained biofilms were dissolved in 2.25 mL 70% ethanol (Sigma-Aldrich). Afterwards, biofilm formation was quantified by directly measuring optical density at 595 nm (OD_595_) in a Spark multimode plate reader (Tecan, Switzerland) in the 24-well plates or by transferring 200 µL of the dissolved crystal violet into a 96-well plate and then measure OD_595_ in an Epoch 2 plate reader (Biotek). Datasets were tested for normality using a Shapiro–Wilk test (*α* = 0.05). The log transformed datasets were analyzed using a Brown–Forsythe one-way analysis of variance (ANOVA, *α* = 0.05) followed by a Dunnett test to correct for multiple comparisons tests. All statistical analyses were performed using Prism v. 9 (GraphPad, USA).

### MIC Determination

To assess the functionality of the constructed β-lactamases in *V. cholerae* (Tables 1 and 2), antimicrobial susceptibility against ampicillin was determined using MIC Test Strips (Liofilchem, Italy). Briefly, a bacterial suspension with an optical density of 0.5 McFarland (1.5 × 10^8^ CFU/mL) units was prepared in 0.9% saline (Sigma-Aldrich). This suspension was then plated on LB agar supplemented with 5 mg/L chloramphenicol before the ampicillin MIC test strip was added. The ampicillin MIC was visually determined after incubation for 20 h at 37 °C.

### Mutagenesis of KPC-2

The KPC-2 mutant library used in this study was constructed using error-prone PCR to introduce mutations in the *bla*_KPC-2_ gene as previously described (Fröhlich et al. 2022). Briefly, the mutational library was constructed by error-prone PCR using 10 ng vector DNA, GoTag DNA polymerase (Promega, USA), 25 mM MgCl_2_ (Promega), 10 μM of primers P7/P8, and either 50 μM oxo-dGTP or 1 μM dPTP. PCR products were *Dpn*I digested for 1 h at 37 °C. Five nanograms of each product was used for a second PCR, which was performed as described above, but without mutagenic nucleotides. The PCR product from the 2nd PCR was digested using *Nco*I and *Xh*oI and ligated into the digested and purified vector backbone. The resulting ligation reaction was transformed into MP21-5 (*E. coli* E. cloni). To ensure that the entire sequence space was sampled, >5,000 mutants were harvested. The mutational library was isolated from *E. coli* E. cloni and transformed into *V. cholerae* C6706 (resulting in MP30-72), and selected on LB plates containing 5 mg/L chloramphenicol. Once again, >5,000 colonies were harvested to ensure that the entire sequence space of KPC-2 was sampled.

### Biofilm Selection and Isolation of Novel KPC-2 Variants

Overnight cultures of MP30-72 were prepared in 3 mL LB medium (*n* = 2) supplemented with 5 mg/L chloramphenicol and incubated overnight at 37 °C with shaking (700 rpm). Cultures were diluted 1:100 in 2 mL LB supplemented with 5 mg/L chloramphenicol in a 24-well plate and incubated statically at 37 °C for 48 h. After 48 h, biofilm pellicles were harvested and transferred into 1 mL phosphate saline buffer (Fisher Bioreagents, USA, 0.137 M NaCl, 0.0027 M KCl, and 0.01 M phosphate, pH 7.4) using a sterile inoculation loop. Afterwards, the suspension was vortexed for 120 s to disintegrate the biofilm and dislodge biofilm-embedded bacterial cells. To isolate single biofilm-evolved clones, 1 μL of the bacterial suspension was spread onto LB agar supplemented with 5 mg/L chloramphenicol and incubated overnight at 37 °C. Single clones were randomly harvested and subsequently Sanger sequenced (Genewiz) to identify genetic changes in KPC-2. Finally, isolated mutants harboring mutations in KPC-2 were subcloned into the original isogenic backgrounds.

## Supplementary Material

evae030_Supplementary_Data

## Data Availability

The data underlying the figures in this article are published alongside the article as source file.
