# Supplementary material for: The Biofilm Lifestyle Shapes the Evolution of β-Lactamases
Source: Genome Biol Evol. 2024 Feb 15;16(3):evae030. doi: 10.1093/gbe/evae030 (PMC10917518; doi:10.1093/gbe/evae030)
Supplement: evae030_Supplementary_Data [file evae030_supplementary_data.zip › Biofilm-lifestyle_Supplementary-information_GBE_finale-without-changes.pdf]

Supplementary Information for

The Biofilm Lifestyle Shapes the Evolution of β-Lactamases

Øyvind M. Lorentzen[1,#], Anne Sofie B. Haukefer[1], Pål J. Johnsen[1], and Christopher Frøhlich[1,#]

[1] Department of Pharmacy, UiT The Arctic University of Norway, Tromsø, Norway

[#] equal contribution; corresponding authors: oyvind.m.lorentzen@uit.no and christopher.frohlich@uit.no

Contents

1. Supplementary tables 2
   Table S1: Area under the growth curves 2
   Table S2: Strains constructed and used in this study. 3
   Table S3: Primers used in this study 4
2. Supplementary figures 5
   Figure S1: Fitness effect of β-lactamases in *V. cholerea*. 5
   Figure S2: Multiple sequence alignment of wtKPC-2 and evolved variants 6
   Figure S3: Fitness effect of KPC-2 variants in V. cholerea. 7
3. References 8

1. Supplementary tables

Table S1: Area under the growth curves

**Strain no.**	**Inserts**	**Area under the growth curves**[a,b]	***N***
30-73	wtVC[c]	1376 ± 9.0	3
30-71	Control	932.8 ± 12.1	6
30-70	wtKPC-2	1251 ± 6.1	14
32-56	TEM-1	745.4 ± 6.6	6
32-57	CTX-M-15	1136 ± 11.2	6
32-58	NDM-1	1001 ± 95	6
32-59	VIM-2	1175 ± 4.2	6
32-53	CMY-2	878.9 ± 28.0	6
32-54	OXA-48	1229 ± 12.9	6
32-55	OXA-163	919.6 ± 36.3	6
30-77	Δ1-48/N136D/M152I/L167P	975 ± 46.3	5
32-13	Δ1-48	988.5 ± 40.6	3
32-07	N136D	1211 ± 26.6	3
30-75	N136K	1256 ± 4.2	8
32-06	L167P	1243 ± 5.0	3

[a] Growth curves are determined in *V. cholerae* C6706
[b] Error is given as the standard error of the mean.
[c] wtVC refers to the parental *V. cholerae* strain C6706.

Table S2: Strains constructed and used in this study.

Strain no.	Strain background [a]	Vector number	Insert	Reference
30-73	C6706	None	None	(Thelin and Taylor 1996)
30-71	C6706	pUNS-233	pA15 vector without *bla* gene	This study
30-70	C6706	pUNS-146.2	KPC-2	This study
32-56	C6706	pUNS-239	TEM-1	This study
32-57	C6706	pUNS-156	CTX-M-15	This study
32-58	C6706	pUNS-157	NDM-1	This study
32-59	C6706	pUNS-236	VIM-2	This study
32-53	C6706	pUNS-158	CMY-2	This study
32-54	C6706	pUNe-4	OXA-48	This study
32-55	C6706	pUNS-178	OXA-163	This study
30-72	C6706	pUNS-146.2	Mutational library of pUN-bla_{KPC-2}	This study
30-65	C6706	pUNS-240	KPC-2: S70A	This study
30-76	C6706	pUNS-241	KPC-2: Δ1-48/N136D/M152I/L167P	This study
30-77	C6706	pUNS-241.1[b]	KPC-2: Δ1-48/N136D/M152I/L167P	This study
30-67	C6706	pUNS-242	KPC-2: Δ1-48/S70A/N136D/M152I/L167P	This study
30-74	C6706	pUNS-238	KPC-2: N136K	This study
30-75	C6706	pUNS-238.1[b]	KPC-2: N136K	This study
30-69	C6706	pUNS-243	KPC-2: S70A/N136K	This study
32-13	C6706	pUNS-245	KPC-2: Δ1-48	This study
32-06	C6706	pUNS-247	KPC-2: L167P	This study
32-07	C6706	pUNS-248	KPC-2: N136D	This study
		**Cloning strains:**		
21-05	*E. coli* E. cloni® 10G	None	None	Lucigen
24-44	*E. coli* E. cloni® 10G	pUNS-146.2	KPC-2 (KU665642; modified)	(Fröhlich et al. 2022)
30-60	*E. coli* E. cloni® 10G	pUNS-239	TEM-1 (NG_050145.1)	This study
24-80	*E. coli* E. cloni® 10G	pUNS-156	CTX-M-15 (NG_048814.1)	(Fröhlich et al. 2022)
24-81	*E. coli* E. cloni® 10G	pUNS-157	NDM-1 (NG_049326.1)	(Fröhlich et al. 2022)
30-57	*E. coli* E. cloni® 10G	pUNS-236	VIM-2 (NG_050347.1)	This study
12-69	*E. coli* E. cloni® 10G	pUNS-158	CMY-2 (NG_048935.1)	(Fröhlich et al. 2022)
21-01	*E. coli* E. cloni® 10G	pUNe-4	OXA-48 (CP033880)	(Fröhlich et al. 2022)
29-27	*E. coli* E. cloni® 10G	pUNS-178	OXA-163 (CP033880; modified)	This study
24-45	*E. coli* E. cloni® 10G	-	KPC-2 mutational library	(Fröhlich et al. 2022)
30-64	*E. coli* E. cloni® 10G	pUNS-240	KPC-2: S70A	This study
30-81	*E. coli* E. cloni® 10G	pUNS-245	KPC-2: Δ1-48	This study
30-66	*E. coli* E. cloni® 10G	pUNS-242	KPC-2: Δ1-48/S70A/N136D/M152I/L167P[c]	This study
32-03	*E. coli* E. cloni® 10G	pUNS-247	KPC-2: L167P	This study
32-23	*E. coli* E. cloni® 10G	pUNS-238.1	KPC-2: N136K	This study
30-69	*E. coli* E. cloni® 10G	pUNS-247	KPC-2: S70A/N136K	This study
32-04	*E. coli* E. cloni® 10G	pUNS-248	KPC-2: N136D	This study
30-81	*E. coli* E. cloni® 10G	pUNS-245	KPC-2: Δ1-48	This study
		**Clinical isolates for strain constructions:**		
K34-7	*Pseudomonas*	-	Carrier of pVIM-2	(Taiaroa et al. 2018)

[a] *V. cholerae* C6706 strain originates from El Tor biotype Inaba

[b] Target gene was subcloned after selection into an isogenic pA15 vector backbone and isogenic *V. cholerae* C6706 strain

Table S3: Primers used in this study

No.	Name		Sequence (5' to 3')	Ref.
3	pUN-NcoI	F	GCTTTCCCATGGATGTTTTTCCTCCTTATGTTAAGCTTACTCAG	(Fröhlich et al. 2022)
4	pUN-XhoI	R	GCTTCTCGAGAAGTGGTTAGCGCGTATTTGTG	
7	preOXAseq	F	GATTACGCGCAGACCAAAACG	(Fröhlich et al. 2022)
8	postOXAseq	R	CCTATTTCCCTAAAGGGTTTATTGAGAATATG	
105	NcoI-VIM-2	F	TTTTTTGGCCATGGGATTCAAACTTTTGAGTAAGTTATTGGTCTATTTGACC	This study
106	XhoI-VIM-2	R	TTTTTCTCGAGCTACTCAACGACTGAGCGATTTGTGTG	This study
115	KPC-2_S70A_LguI	F	TTTTTGCTCTTCTGTGCGCGTCATTCAAGGGCTTTCTTGC	This study
108	KPC_2_S70A_LguI	R	TTTTTGCTCTTCGCACAGTGGGAAGCGCTCC	This study
117	KPC-ORF2-NcoI	F	GCTTCCATGGGAGATACCGGCTCAGGCGCAAC	This study
		R	Primer number 4	(Fröhlich et al. 2022)
118	KPC-N136D-LguI	F	TTTTTGCTCTTCGCCGCCGCCGATTTGTTGCTGAAGG	This study
118	KPC-N136D-LguI	R	TTTTTGCTCTTCGCGGCGTTATCACTGTATTGC	This study
119	KPC-L167P-LguI	F	TTTTTGCTCTTCCTGGGAGCCGGAGCTGAACTCC	This study
119	KPC-L167P-LguI	R	TTTTTGCTCTTCCCCAGCGGTCCAGACGG	This study
90	NcoI-TEM-1	F	TTTTTTCCATGGGAAGTATTCAACATTTTCGTGT	This study
91	XhoI-TEM-1	R	TTTTTTCTCGAGTTACCAATGCTTAATCAGTG	This study
54	OXA-163-LguI-	F	TTTTTGCTCTTCTATTCGGGCTAAAACTGGATACGATACTAAGATTGGCTGG	This study
54	OXA-163-LguI	R	TTTTTGCTCTTCGAATAATATAGTCGCCATTG	This study

2. Supplementary figures

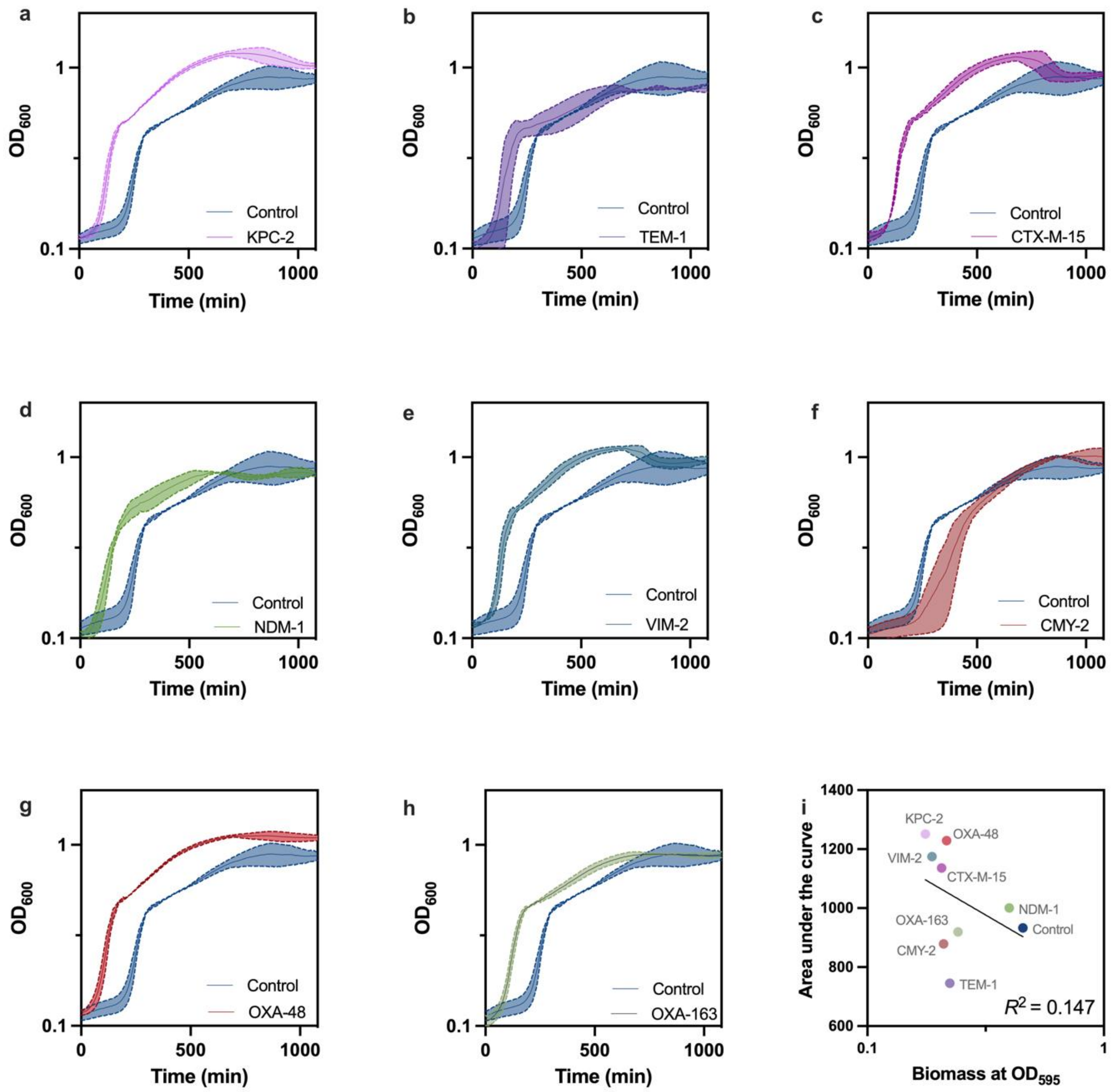

Figure S1: Fitness effect of β-lactamases in *V. cholerae*.

Effect of class A: KPC-2 **(a)**, TEM-1 **(b)**, CTX-M-15 **(c)**, class B: NDM-1 **(d)**, VIM-2 **(e)**, class C: CMY-2 **(f)** and class D: OXA-48 **(g)**, OXA-163 **(h)** β-lactamase and the empty vector control (n = 6) on bacterial fitness in *V. cholerae*. Bacterial fitness was assessed as the area under the growth curve over 18 h of incubation at 37°C. **i.** Assessment of correlation between area under the curve values and the strains' ability to form biofilms (see Tab. 1) resulted in a statistically non-significant Pearson correlation (R^2 = 0.147, P = 0.31). Error around the growth curves represents the standard error of the mean determined by at least 3 biological replicates. See table S1 for overview of N for each strain.

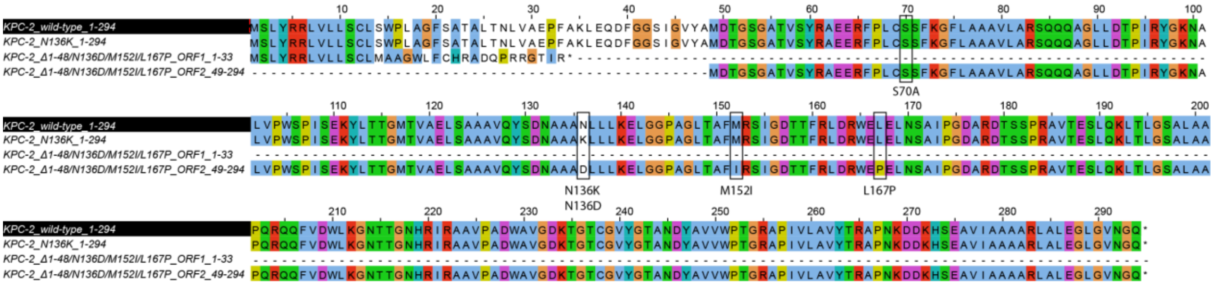

Figure S2: Multiple sequence alignment of wtKPC-2 and evolved variants

Both reading frames of Δ1-48/N136D/M152I/L167P are presented; the first reading frame (Δ1-48/N136D/M152I/L167P_ORF1) includes amino acids 1-33. The nucleotide deletions in nucleotide positions 40 and 41 (amino acid position 14) led to a frameshift which subsequently introduced a premature stop codon. For the second reading frame (Δ1-48/N136D/M152I/L167P_ORF2), an already existing methionine at position 49 was recruited and served as a new start codon for the production of amino acids 49-294. The second open reading frame, comprising amino acids 49-294, is representative for our Δ1-48 mutant. Amino acid substitutions found in Δ1-48/N136D/M152I/L167P (N136D/M152I/L167P) and N136K (N136K), and the active site S70, are shown in brackets at their respective positions. The coloured residues are indicated as follows; Blue: hydrophobic. Red: positive charge. Magenta: negative charge. Green: polar. Orange: glycines. Yellow: prolines. Cyan: aromatic. White: not conserved.

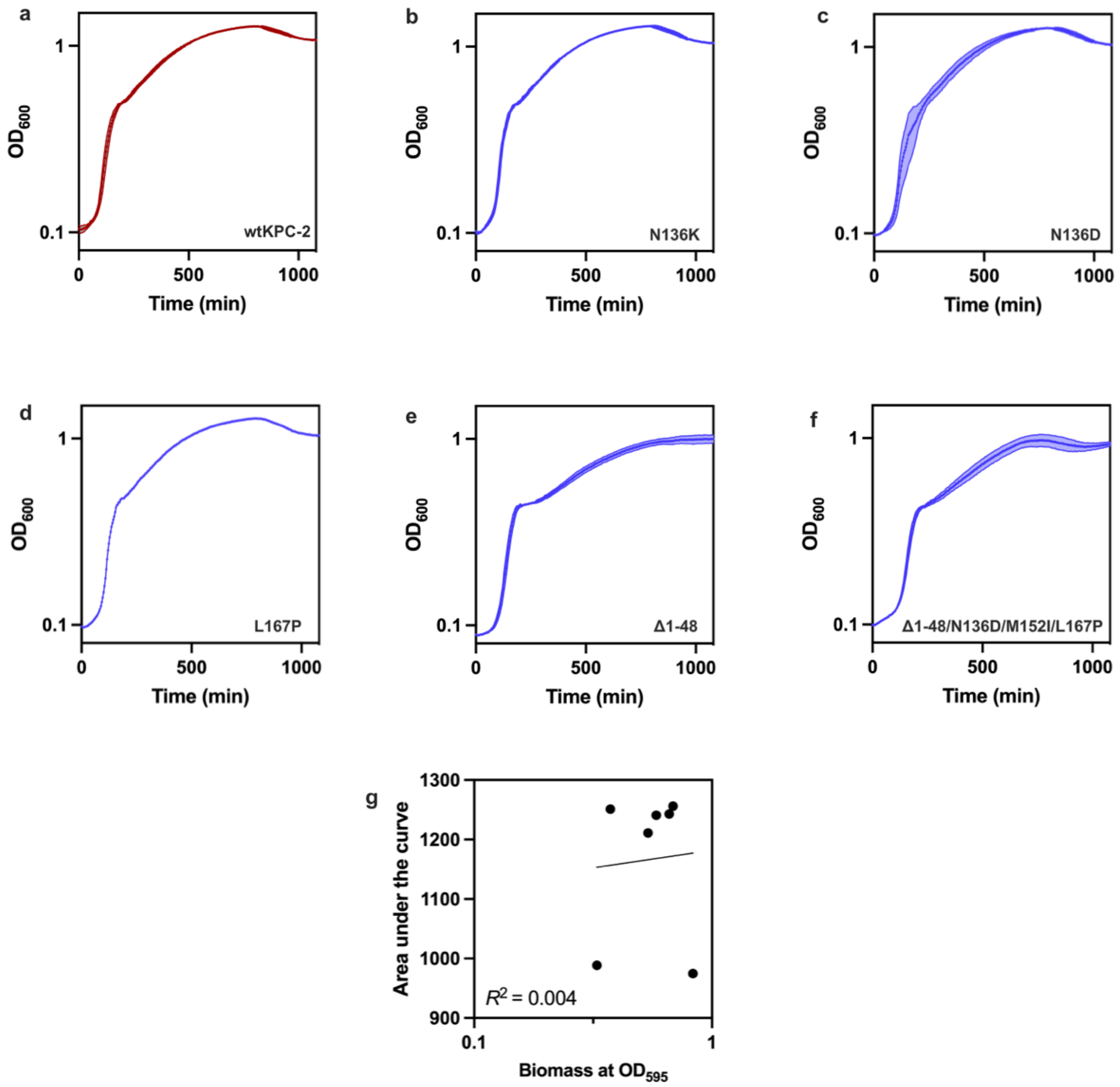

Figure S3: Fitness effect of KPC-2 variants in *V. cholerae*.

Growth curves of different KPC-2 mutants in *V. cholerae*. **a**. wild-type KPC-2, **b**. N136K, **c**. N136D, **d**. L167P, **e**. Δ1-48, **f**. Δ1-48/N136D/M152I/L167P and **g**. the non-significant correlation between area under the curve and the strains' biofilm biomass (see Tab. 2) (Pearson correlation, R^2 = 0.04, P = 0.89). Error around the growth curves represents the standard error of the mean determined by at least 3 biological replicates. See table S1 for overview of N for each strain.

3. References

Fröhlich C, Sørum V, Tokuriki N, Johnsen PJ, Samuelsen Ø. 2022. Evolution of β-lactamase-mediated cefiderocol resistance. *Journal of Antimicrobial Chemotherapy* 77:2429–2436.

Taiaroa G, Samuelsen Ø, Kristensen T, Løchen Økstad OA, Heikal A. 2018. Complete Genome Sequence of *Pseudomonas aeruginosa K34-7*, a Carbapenem-Resistant Isolate of the High-Risk Sequence Type 233. *Microbiol Resour Announc* 7:10.1128/mra.00886-18.

Thelin KH, Taylor RK. 1996. Toxin-coregulated pilus, but not mannose-sensitive hemagglutinin, is required for colonization by *Vibrio cholerae* O1 El Tor biotype and O139 strains. *Infect Immun* 64:2853–2856.
